# Libyan Healthcare Professionals’, Patients’ and Caregivers’ Perceptions and Religious Beliefs about Cancer Pain and its Management: A Descriptive Qualitative Study

**DOI:** 10.1007/s10943-023-01763-1

**Published:** 2023-02-22

**Authors:** Salim M. Makhlouf, Shenaz Ahmed, Michael I. Bennett

**Affiliations:** School of Medicine, Academic Unit of Palliative Care, LIHS, Leeds Institute of Health Sciences, University of Leeds, Level 10 Worsley Building, Clarendon Way, Leeds, LS2 9NL UK

**Keywords:** Cancer pain management, Healthcare professionals, Patients, Caregivers, Religion, culture, perceptions, Healthcare system, Developing countries, Libya

## Abstract

Cancer pain remains a significant problem worldwide. It is often undertreated and presents in about half of cancer patients. Although several guidelines and pharmacological interventions for cancer pain management (CPM) exist, inadequate assessment and undertreatment of cancer pain are well-documented globally, especially in developing countries, including Libya. Perceptions, cultural and religious beliefs of healthcare professionals (HCP), patients, and caregivers about cancer pain and opioids are reported as barriers to CPM globally. This qualitative descriptive study aimed to explore Libyan HCPs’, patients’, and caregivers’ views and religious beliefs about CPM and involved semi-structured interviews with 36 participants: 18 Libyan cancer patients, 6 caregivers, and 12 Libyan HCPs. Thematic analysis was used to analyse the data. Patients, caregivers, and newly qualified HCPs were concerned about poor tolerance and drug addiction. HCPs perceived a lack of policies and guidelines, pain rating scales, and professional education and training as CPM barriers. Some patients were unable to pay for medicines if they faced financial difficulties. Instead, patients and caregivers emphasised religious and cultural beliefs for managing cancer pain, including the use of the Qur’an and cautery. Our results suggest that religious and cultural beliefs, lack of knowledge and training in CPM among HCPs, and economic and Libyan healthcare system-related factors negatively affect CPM in Libya.

**Supplementary Information:**

The online version contains supplementary material available at 10.1007/s10943-023-01763-1.

## Introduction

Cancer pain is a significant problem for many cancer patients worldwide because it is often associated with adverse effects on people’s Quality of Life (QoL), including physical, social, psychological, and financial well-being (Ferrell, 1995). The prevalence of cancer pain remains high, and its symptoms are distressing (Fischer et al., 2010; Lemay et al., 2011). A meta-analysis conducted in the USA included 169 studies on cancer pain and reported that more than half of patients who received anticancer treatment and about two-thirds of cancer patients in advanced stages experienced moderate to severe pain (van den Beuken-van Everdingen et al., 2016). In Arab countries, the prevalence of cancer pain was reported by cancer patients in approximately 73% of a convenience sample of 162 Jordanian patients (Al Qadire et al., 2013), 9% of Saudi cancer patients (Kaki, 2006), and 40% of 400 Lebanese cancer patients (Hamieh et al., 2018). No data were found regarding the prevalence of cancer pain in Libya. Although several guidelines and pharmacological interventions have existed for cancer pain management (CPM) (NICE, 2012; WHO, 1996, 2019), inadequate assessment and undertreatment of cancer pain are well-documented worldwide (Greco et al., 2014; Li et al., 2018; Mori et al., 2012; Thinh et al., 2018). A systematic review of 20 articles reported that about one-third of patients with cancer pain did not receive adequate CPM (Greco et al., 2014). A survey involving 162 Jordanian cancer patients reported that approximately 30% of patients with cancer pain had not been treated for their pain (Al Qadire et al., 2013). Another survey conducted in Lebanon included 400 cancer patients, which showed that inadequate CPM was found in about 47% of cancer patients (Hamieh et al., 2018).

Opioid analgesics, such as morphine, remain the most effective method/treatment recommended for CPM (WHO, 2019) and are frequently used/accessible in the most developed countries. However, studies in developing countries have reported that access to such opioids for CPM is either limited or legally restricted (Shamieh & Jazieh, 2010). In developing countries, including Libya, opioids are not frequently used, and this may be because access to strong opioids is challenging, and their use may be rejected for CPM by some HCPs, patients, and caregivers due to personal perceptions and views about opioids, which could be related to their religious or cultural beliefs (Cleary et al., 2013). Therefore, there is evidence that cancer patients in developing countries still do not receive appropriate CPM (INCB, 2019; Saini & Bhatnagar, 2016). This issue is evident in Libya, as between 2014 and 2018, no data were available regarding the consumption of opioids, such as morphine, for palliative care and CPM in Libya (INCB, 2019). A similar study in Egypt concluded that low consumption figures of opioids such as morphine indicate inadequate CPM (Alsirafy, 2010).

Many barriers to effective CPM exist, including HCPs, patients, caregivers, and healthcare system-related factors. For example, negative attitudes and lack of knowledge towards CPM among HCPs, patients, and caregivers, and a lack of policies and CPM guidelines in health systems were reported as barriers to adequate CPM (Kwon, 2014; Makhlouf et al., 2020, 2022; Oldenmenger et al., 2009; Potter et al., 2003). Furthermore, religious and cultural beliefs could be barriers to effective CPM. It has been stated that cultural and religious beliefs can affect patients’ interpretation of their pain and consideration of treatment (Colak et al., 2014; Silbermann & Hassan, 2011). For instance, many patients and their caregivers believed that the Qur’an and the prayers could cure diseases, such as cancer, and relieve physical suffering like pain (Erol et al., 2018; Hatamipour et al., 2015; Hosseini et al., 2016; Makhlouf et al., 2020). Thus, some patients and their caregivers usually prefer to use the Qur'an and prayers instead of medical medications to help the patients cope with their disease, anxiety, and pain (Ibrahim et al., 2020; Pathmawathi et al., 2015). Our recent systematic review found that patients’ and caregivers’ perceptions of cancer pain and opioids are influenced by their religious and cultural beliefs (Makhlouf et al., 2020). For instance, studies with Turkish and Jordanian patients show that they reject strong opioids for CPM because of their negative attitudes towards them, and in particular, they fear drug addiction citing religious and cultural reasons for rejecting such opioids for CPM (Al Qadire, 2012; Colak et al., 2014).

Some cancer patients and their caregivers may develop misconceptions about CPM depending on their cultural background. One study found that American Indian patients, caregivers, and HCPs believed that expressing pain was seen as a sign of weakness, and complaining about cancer pain will only extend their vulnerability (Haozous & Knobf, 2013). Another study showed that Taiwanese cancer patients do not report cancer pain and refuse to use opioids as, in their culture, they consider pain a necessary aspect of life (Chou et al., 2011).

The cautery is one such traditional therapy used by some patients and their caregivers in Arabic countries for cancer and pain management, which should be discouraged (Abou-Elhamd, 2009). The ancient Egyptians had great faith in the therapeutic values of fire; thus, they used cautery to stop bleeding (Abou-Elhamd, 2009). The cautery involves creating burns on the tissue (by overheating a knife or piece of iron using the fire) to either close wounds or stop bleeding because the heat would make the blood clot or remove part of the body (Al Binali, 2004). Choosing the location for applying the cautery depends on the patient's complaint (i.e. type of disease or pain) (Al Binali, 2004). For instance, in cases of diabetic foot, the cautery is commonly applied to the dorsum of the patient’s foot or the lateral aspect of his lower leg (Al-Wahbi, 2006). Whereas in the care of jaundice, it is applied to the patient’s left hand. For treating sciatica, cautery could be used in up to 17 different locations of the body. If the patient complains of chest pain with shortness of breath (i.e. angina or myocardial infarction), cautery can be applied to the 4th and the 5th anterior or posterior ribs of the patient's body in the same location of pain (Al Binali, 2004). Cautery may act in the same way as acupuncture, stimulating the release of endogenous opioids and other neurotransmitters, which can prevent the feeling of pain (Abou-Elhamd, 2009). In Arab countries, including Libya, some patients and caregivers prefer to use cautery (Kaiy—ironing the place of cancer or pain with fire) as alternative therapy instead of medical treatments for cancer and pain management (Aboushanab & Alsanad, 2018; Farid & El-Mansoury, 2015).

It seems that the preference for using cautery by some patients and their caregivers to manage cancer and pain might be based on a specific narration narrated by the Prophet Muhammed (PBUH). However, there was probably a misunderstanding about this narration, as the authentic narration is that the Prophet (PBUH) *said: “Healing is in three things: A gulp of honey, cupping and branding with fire (cauterising). Nevertheless, I forbid my followers to use (cauterisation) branding with fire.”* (Al-Bukhari, 1996 cited in; Fitzpatrick & Walker, 2014, p. 264). Unfortunately, because of the misinterpretation of this narration, some patients and their caregivers might prefer to use cautery (Kaiy) instead of medical treatment to manage their cancer and pain. Many studies indicated that cultural beliefs, including the use of cautery, had an adverse effect on CPM (Abou-Elhamd, 2009; Farid & El-Mansoury, 2015). For example, Farid and El-Mansoury (2015) stated that due to the application of cautery by Libyan patients to cure their cancer, cancer management is usually delayed, increasing the aggressiveness of the disease. Due to religious and cultural beliefs, some patients believe that they should endure their pain courageously (Colak et al., 2014; Ho et al., 2013). Therefore, it is possible that religious and cultural beliefs can be barriers to effective CPM.

Evidence suggests that HCPs should recognise and understand that patients often refer to their religious, spiritual, and cultural beliefs when considering medical treatment (Swihart & Martin, 2020). Understanding patients’ religious and cultural beliefs can enable HCPs to improve CPM, as such beliefs should guide HCPs in how and when patients’ pain should be treated (Silbermann & Hassan, 2011). A recent survey showed that Libyan HCPs are concerned about the social stigma of opioids due to a fear of poor tolerance and drug addiction, which could be related to their religious and cultural beliefs, resulting in barriers to effective CPM (Makhlouf et al., 2022). Similar findings also were found in other countries (Kagawa-Singer, 2011; Nasser et al., 2016; Rajeh Saifan et al., 2019). A survey reported that about 45% of Lebanese physicians hesitated to prescribe strong opioids, such as morphine, for CPM due to the social stigma of opioids, including fear of side effects, poor tolerance, and drug addiction (Nasser et al., 2016).

Given the similarities in religious and cultural practices between Libya, Jordan, Turkey, Saudi Arabia, and Lebanon, Libyan HCPs, patients, and their family caregivers' perceptions, beliefs, and negative attitudes may also be similar to those described in the Jordanian, Turkish, Saudi, and Lebanese studies (Al Qadire, 2012; Colak et al., 2014; Kaki, 2011; Nasser et al., 2016). However, in Libya, cancer pain is more likely to be under-measured and under-treated than in those countries mentioned earlier because of underfunding and the collapse of the Libyan healthcare system since the revolution in 2011 (El Oakley et al., 2013). Furthermore, Libya lacks healthcare services, such as pain management (Elzahaf et al., 2016; Petropoulos et al., 2016) and palliative care (El Ansary et al., 2014). A recent survey reported that Libyan HCPs had poor attitudes towards opioids and lacked knowledge about CPM (Makhlouf et al., 2022). Many studies indicated that HCPs, who have experience in pain clinics and palliative care settings, showed better attitudes towards opioids and knowledge about CPM than those who did not (Darawad et al., 2019; McCaffery & Ferrell, 1995; Rurup et al., 2010).

Moreover, knowledge about potential barriers to effective CPM due to perceptions, beliefs, and attitudes was documented in many studies from different countries (Al-Ghabeesh et al., 2020; Kwon, 2014; Makhlouf et al., 2020). However, there is no published research on this subject in Libya. In addition, the CPM situation among Libyan oncology HCPs, cancer patients, and caregivers has not been previously assessed, despite the poor QoL, which has been found among cancer patients in Libya (Agila, 2020; Hashemi et al., 2019; Nouh et al., 2018). Accordingly, this study aims to explore Libyan HCPs’, patients’, and caregivers’ views and religious beliefs about cancer pain and its management.

## Methods

### Study Design

The study received ethical approval from the University of Leeds, School of Medicine Research Ethics Committee (MREC 18-064). The authors faced a challenge while selecting a qualitative design for this study, as at the beginning, they thought phenomenology would be an appropriate design. However, after extensive reading of the literature and consulting with qualitative researchers, the authors found that none of the well-known qualitative research designs, including ethnography, phenomenology, grounded theory, narrative, and case study (Chase, 2011; Creswell, 2007; Moran, 2002; Neuman, 2011), were suitable to achieve the aim of this study. Hence, a qualitative description was chosen for the research design of this study because it seeks to discover and understand a specific phenomenon, a process, or the perspectives and worldviews of the individuals involved (Caelli et al., 2003; Merriam, 1998). Although the qualitative descriptive design is considered less interpretative than other forms of qualitative designs (e.g. phenomenology), it is not free of interpretation and produces findings close to the data (Sandelowski, 2010). It has been stated that the use of a qualitative descriptive design is appropriate where information is required directly from people who are experiencing a specific phenomenon under target investigation (Bradshaw et al., 2017; Neergaard et al., 2009). The fundamental aspect of the qualitative descriptive design is valuable in its own right (Bradshaw et al., 2017). The most frequently proposed rationale for using a descriptive design is to provide a straight descriptive summary of experiences and perceptions (Sandelowski, 2010), mainly in areas where little is known about the phenomenon of investigation. This is in line with the aim of this study. The participants' perceptions were recorded involving face-to-face, semi-structured interviews.

### Sampling and Recruitment

Purposive sampling was used in this study to recruit individuals who have experience with cancer pain and CPM. Libyan cancer patients, who were seeking oncology treatment at an oncology centre in Egypt, their family caregivers, and Libyan HCPs (oncologists and oncology nurses) who were having training courses in oncology, were recruited through either a receptionist or an oncology nurse at an oncology centre in Alexandria, Egypt. Inclusion criteria were adults over 18 from the following groups; Libyan oncology doctors and nurses, advanced cancer patients, and caregivers. Patients eligible to participate had been diagnosed with advanced cancer at stages (II & III and IV), associated with pain based on their hospital records, and patients who were waiting for chemotherapy or radiotherapy or waiting to see their doctors. Staff at this site (oncologist practice manager and oncology nurse) screened patients’ records to identify if patients were meeting the eligibility criteria for this study. Caregivers were adults who had been caregivers for a minimum of 3 months and travelled with their patients to Egypt for treatment; HCPs had worked in a Libyan oncology setting for more than six months. Only participants who could give written consent to participate and without communication difficulties were included.

### Data Collection

The first author (S.M., bilingual in English and Arabic) collected data via face-to-face semi-structured interviews. All interviews were taken individually in a private and quiet room for participants’ privacy and confidentiality at the SUN oncology centre, Egypt. The interviews were conducted in Arabic and lasted approximately 30 to 45 min. A semi-structured interview was chosen as it combines the strengths and eliminates the weaknesses of structured and unstructured interviews, and it is the most commonly used approach in health research for qualitative data gathering (Gill et al., 2008). Furthermore, such an approach enables an in-depth understanding of the participant's perception or experience of one target research subject (Britten, 1999; Gill et al., 2008). Interviews were audio-recorded. Semi-structured interview topic guides were developed based on the current literature, and our systematic review (Makhlouf et al., 2020), and the study aims to guide the researchers and ensure consistency (Hadi et al., 2017). Demographic information was also collected for all participants. A professional transcriber transcribed all the interviews in Arabic. All interviews took place in Alexandria at an oncology centre in Egypt between June and September 2019. To avoid or minimise issues that might arise during data collection, it was essential to conduct a pilot study as it helps refine the elements of instruments required for data collection (Bryman, 2016). This test also helps to ensure the questions included in the research instruments are reorganised and straightforward for both the researcher and target participants (Saunders et al., 2016). Pilot testing questions were included in this study.

In qualitative studies, the sample size is typically small, focusing on the volume and richness of information collected (Patton, 2002). According to Patton (2002), there is no rule for sample size in qualitative studies. Thus, the sample size can be based on *“what you want to know, the purpose of the study, what is at stake, what will be useful, what will have credibility, and what can be done within the available time and resources.”* Creswell (2013) stated that the ideal sample size figure for a qualitative study should range from 5 to 30 based on the type of approach that a study follows (e.g. phenomenology or grounded theory). A total of 41 participants were approached; 5 patients were excluded as they were diagnosed with early-stage cancer. No participants declined, resulting in a total of 36 participants that met the inclusion criteria (Corbin & Strauss, 2008).

The sample size in qualitative studies can be determined by information saturation, which means that the sampling will be terminated when no more new information is forthcoming, and therefore, redundancy will be the primary criterion (Bryman, 2004; Lofland & Lofland, 1995). The concept of “saturation”, which is borrowed from grounded theory, has been used by many qualitative researchers to assess whether or not the sample size is proper in a qualitative study (Malterud et al., 2016; Sandelowski, 1995). Hence, the sample size of this qualitative study was 36 participants, which was more than sufficient as the interviews were in-depth, face-to-face, and semi-structured. A sample size of more than 36 participants was not accepted as time-consuming for data analysis as the analysis usually takes more time, and more participants would not contribute to the effectiveness of the study. This study's analysis of these 36 transcripts revealed that data saturation had been reached and recruiting further participants was unnecessary; more new qualitative data would produce redundant information (Fusch & Ness, 2015). To avoid bias and ensure the consistency and reliability of collected information, the same first investigator conducted semi-structured face-to-face interviews with Libyan patients, caregivers, and HCPs, and the findings were reviewed by the other two authors (Morse et al., 2002). For additional lending credibility to our data, data gathering and analysis were thus simultaneous and iterative (Giacomini & Cook, 2000; Mays & Pope, 2000).

## Data Analysis

The first author (S.M.) analysed the data using thematic analysis (Sandelowski, 2010). To guide, identify, and interpret patterns of meaning within the qualitative data analysis, a six-step process proposed by Braun and Clarke was used (Braun & Clarke, 2006). A deductive approach was initially employed to organise and analyse data according to themes identified a priori. Deductive coding was utilised in which categories from earlier literature shaped the interview guide and initiated the categories in which the data were placed. During the coding process, patterns were identified across all the items of data and over time. The final refined themes were deductively analysed to develop a thematic map, which demonstrates the interaction of themes and provides a more complete inquiry (Morse, 2020). This was followed by an inductive approach to identify emerging themes. Line-by-line coding was applied to code individual transcripts, and the coding outline was checked independently by two authors for validity, one of whom was an experienced qualitative researcher (S.A.). Once duplicate codes were eliminated and relevant data emerged from individual interviews, the researchers searched for potential themes. Similar themes were combined; other themes were either divided or renamed if needed. The thematic map was developed iteratively from initial diagramming from reflections on the data and through critical discourse with the research team in the light of the data and previous research in the current area. Subsequent meeting was convened between the research team and independent reviewers to discuss any possible discrepancies in data analysis and interpretation. This commenced at the coding stage to challenge the inductive interpretation of the data and concluded with the final version of the deductive thematic map. Such meetings were done to challenge interpretation and reinterpretation, as well as to deepen understanding (Tracy, 2010).

Data analysis also involved consistent cross-referencing between the participants for comparisons based on gender, age, and educational status. NVivo 12 Plus was used to analyse the Arabic transcripts; these were not translated into English to avoid any meaning loss through translation and ensure reliability and validity (Twinn, 1997). The first author (S.M.) analysed the data and wrote his interpretation of it in English. Key quotes to support this interpretation were translated into English. To increase our results’ dependability, the first researcher (S.M.) described and discussed detailed themes with the other two authors (S.A. and M.B.) (Lincoln & Guba, 1986), then refined and discussed them again to ensure consistency in the interpretation of the data. To ensure rigour and trustworthiness of study findings, debriefing and providing "thick description" methods were utilised (Green & Thorogood, 2018; Shenton, 2004). The data source triangulation technique was applied by using several groups of oncology department staff working in different Libyan oncology settings with different roles to strengthen our results regarding confirmability and credibility (Shenton, 2004). Furthermore, the questions for the interviews were piloted to increase the credibility of this study. Moreover, to help reduce the interview bias, and to assist the researcher in avoiding asking leading questions, which can lead to bias in the answers, the interview schedule contained prompts (Kvale, 2007), and the interview transcripts’ quotes were used in the findings and presented transparently.

Additionally, semi-structured interview topic guides were developed based on the existing literature and our systematic review (Makhlouf et al., 2020), and the study aims to guide the researchers and ensure consistency (Hadi et al., 2017). Besides, to avoid bias and to ensure the consistency of collected information, the same investigator (SM) conducted semi-structured face-to-face interviews with Libyan patients, caregivers, and HCPs, and the findings and themes were reviewed by the other two authors (SA and MB) (Morse et al., 2002). To ensure transferability, the researchers provided a detailed and profound description of the study findings to help readers use the relevant results in their context. Study methodology and methods, including study design, sampling and recruitment, study settings, data collection, and analysis, were also detailed to enhance transferability (Willig, 2013). The Consolidated Criteria for Reporting Qualitative research (COREQ) criteria were applied to maintain research quality and guide the reporting of the findings in this study (Tong et al., 2007)

## Results

Thirty-six participants were interviewed: 12 Libyan HCPs, 18 patients, and 6 caregivers. See Table 1 for participants’ demographic characteristics.

**Table 1:** Participant’s demographic characteristics

	HCPs (*n* = 12)	Patients (*n* = 18)	Caregivers (*n* = 6)
Gender *n* (%)			
Male	5 (41.66)	9 (50)	6 (100)
Female	7 (58.33)	9 (50)	0 (0)
Age (years)			
Mean (SD)	37.25 (9.51)	48.5 (14.39)	37.5 (12.83)
Range	22–50	21–75	28–60
Marital status *n* (%)			
Married	6 (50)	12 (66.66)	3 (50)
Single	4 (33.33)	4 (22.22)	3 (50)
Divorced	1 (8.33)	2 (11.11)	0 (0)
Widowed	1 (8.33)	0 (0)	0 (0)
Education *n* (%)			
Elementary	0 (0)	5 (27.77)	0 (0)
Standard	2 (16.66)	1 (5.55)	2 (33.33)
Intermediate	4 (33.33)	9 (50)	1 (16.66)
Undergraduate	3 (25)	2 (11.11)	3 (50)
Postgraduate	3 (25)	1 (5.55)	0 (0)
Profession *n* (%):			
Nurses	6 (50)		
Physicians	6 (50)		
Monthly salary			
Range; (£/month)	< 500–1500	< 500	< 500
No income *n* (%)	0 (0)	5 (27.77)	1 (16.66)
Cancer diagnosis *n* (%)			
Breast		5 (27.77)	
Lung		2 (11.11)	
Pancreatic		1 (5.55)	
Nasopharyngeal		1 (5.55)	
Lymphoma		2 (11.11)	
Bladder		2 (11.11)	
Stomach		2 (11.11)	
Colorectal		3 (16.66)	
Stage of cancer *n* (%)			
II &III		12 (66.66)	
IV		6 (33.33)	
Type of pain medication *n* (%)			
None		13 (72.22)	
On pain medication		5 (27.77)	
Paracetamol		2 (11.11)	
NSAID		3 (16.66)	
Codeine		0 (0)	
Tramadol		0 (0)	
Morphine		0 (0)	

*HCPs =* healthcare professionals, *n* = number, *SD* = standard deviation, % = percentage, / = per, *£* = pound sterling, <  =  less than

**Fig. 1:**
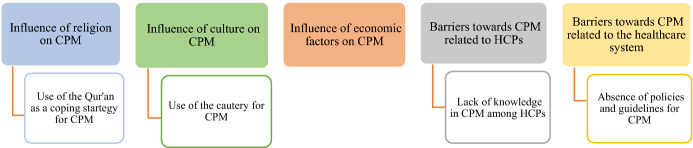
Thematic map: themes and sub-themes

Five themes were identified, including the influence of religious, cultural, and economic factors on CPM, and barriers related to HCPs and the healthcare system, as illustrated in Fig. 1.

## Influence of Religion on CPM

### Use of the Qur’an as a Coping Strategy for CPM

The Qur'an was often used by patients, caregivers, and some HCPs to help patients cope with their disease, anxiety, and pain. Some patients relied on reciting verses from the Qur’an to support themselves in managing their condition and specifically their cancer pain. Similarly, the majority of caregivers said that they often used the Qur'an to relieve the patient’s distress that could be associated with cancer and pain. Some caregivers preferred to use the Qur'an instead of painkillers for CPM. This could be why some Libyan patients and their caregivers refused to use opioids for CPM in the current study:

*“…… When I feel pain, I do not use pain medications. To relieve my pain, after each prayer, I put my hand on the place of the pain and recite specific Ayahs (Verses) from the Qur'an. That makes me feel better and relaxed because this reciting helps me endure my pain. For instance, as Allah says in the Qur'an (And We send down of the Qur'an that which is a cure and a mercy for the believers)”* Verse (17:82 Surat AL-Isra)*.* P10

*“As Muslims, people usually are positively affected by the Qur'an and the prayer, so they commonly use them as supportive strategies alongside the treatment to cope with cancer and pain. For example, we usually use Al-Ruqyah Al-Shariah (i.e., reciting the Qur'an on the water, then the patient drinks it). We also read or listen to the Qur'an and do prayer to support our patients; after that, the patient usually feels peaceful and relaxed. Accordingly, I do not prefer my brother to use pain medications like morphine for CPM...”* C5

Similarly, some HCPs reported the use of verses from the Qur’an alongside medical treatment as a coping strategy to help and support their patients in coping with their cancer and pain. They believed such a strategy usually helped their patients tolerate or minimise distress associated with stressful procedures during cancer and pain treatments. However, they (HCPs) did not believe that Qur’an could cure cancer and relieve cancer pain. This finding indicates that the use of the Qur’an could help to distract patients from their disease and anxiety:

*“………, we usually use different treatment methods, including medication and religious support. For example, Allah says in the Qur'an (O you who have believed, seek help through patience and prayer. Indeed, Allah is with the patients), verse (2:153 Surat L-Baqarah). This verse can be used to comfort and support the patient during his/her treatment. Nevertheless, we usually advise patients to take their pain medications for cancer pain, as some patients and their caregivers usually prefer to use religious beliefs only as a coping strategy to cope with cancer pain. In this case, we as the medical team cannot do anything as this is their wishes and choice.”* D1

Relying exclusively on religious beliefs to cope with pain can be a barrier to CPM because some patients and their caregivers might reject pain medications because they believe the Qur’an and prayer will relieve such suffering. Although several patients and their caregivers were satisfied with using the Qur’an and prayer to manage cancer pain, this could influence how some patients and caregivers refused to use opioid analgesics for managing pain with cancer.

## Influence of Culture on CPM

### Use of the Cautery (Kaiy) for CPM

Many patients and their caregivers discussed cautery (Kaiy—ironing the place of cancer or pain with fire) as an alternative therapy for CPM. For example, some patients and their caregivers believed that cautery was the faster way to treat the disease and pain. Therefore, they preferred to use it instead of medical CPM. The majority of HCPs acknowledged that a lack of education contributed to patients and their caregivers’ preference for cautery as an alternative therapy to CPM:

*“……...when I was diagnosed with cancer, I did use the Kaiy, as my family took me somewhere (not a clinical place) to do it,.……...after that, I felt comfortable, as the pain has gone. Thus, I have not used painkillers anymore, and I prefer to use Kaiy for my cancer pain.” P15*

*“Some caregivers prefer to use the Kaiy to manage their patients’ cancer and pain instead of medicine, as it can be done in one time and faster. After applying the Kaiy, the patients and their caregivers feel relaxed as they believe it can treat cancer and relieve pain. Thus, family caregivers usually convince their patients to do so and leave the hospital as the last option.” C4*

*“…..., our community is affected by their cultural beliefs, especially uneducated people. Therefore, some of them usually use cautery instead of medical treatment before coming to the hospital. This results in a delay in cancer and pain management. For example, some patients often come with advanced cancer and severe pain, as they tried alternative medicine (e.g., Kaiy) before they came to the unit.” D2*

Many HCPs believed that a lack or low level of education contributed to cancer patients and their caregivers’ preference for cautery as an alternative therapy to CPM. These results imply that cultural beliefs (i.e. the use of cautery) could be a barrier to effective CPM, especially for poorly educated patients and caregivers. Thus, it can be suggested that the use of cautery is another issue that influences CPM in Libya.

## Influence of Economic Factors on CPM

Financial difficulties may also be a barrier to CPM. For example, most patients said they had experienced financial hardships due to cancer procedures and treatments. Some patients reported selling their properties and borrowing money to pay for their cancer treatments. Similarly, some caregivers with difficult financial circumstances emphasised that cancer-related costs had an impact on the whole of their families:

*“……. I did not use painkillers for my cancer pain, and I endured my pain as we spent all the money on cancer treatment, so I could not afford to pay for painkillers. My husband had to sell his car and borrowed money to pay for cancer treatment, food, travel, and accommodation expenses.”* P3

*“….. the cost of cancer treatment and stay at a private hospital were expensive. Thus, we did struggle to afford full money. For example, we did pay for the operation of about 27.000 Libyan Dinars. Furthermore, the cost of cancer treatment, travel, hotel, and food. All these costs overburdened us.”* C5

This consequence could lead to some patients might experience cancer pain, and they had to endure their pain untreated, as they might not have enough money to pay for such medication, or they may not want to bother their families about extra costs related to pain medication for their CPM.

## Barriers to CPM Related to HCPs

### Lack of Knowledge in CPM Among HCPs

Most HCPs believed they lacked comprehensive knowledge of CPM and attributed this to an absence of CPM training for HCPs. Thus, they believed that managing patients with cancer pain was a challenge. Other doctors said that they used self-education and personal experience in cancer care settings for CPM:

*“……. As oncology doctors and nurses, we do not have enough knowledge of CPM, as nobody in our team has training in CPM. Therefore, some professionals usually focus on treating the disease itself. Few doctors sometimes use textbooks and internet resources for CPM and their daily work experience in a cancer care setting. However, many doctors and nurses do not often pay attention to cancer pain.”* D3

*“…… In Libya, many nurses lack knowledge and experience in CPM, as we did not have training or education courses in CPM. Hence, nurses usually do not prefer to talk about CPM. Libyan nurses generally have a limited duty, and CPM is not a part of our duty. Nurses in Libya only do the doctors’ requests.”* N5

Accordingly, managing patients with cancer pain was a challenge that HCPs could face in Libyan hospitals. Some Libyan physicians used their self-education and personal experience in cancer care settings to manage patients with cancer pain. This outcome indicates that CPM might be inadequately managed as many HCPs showed a lack of knowledge about CPM.

## Barriers to CPM Related to the Healthcare System

### Absence of Policies and Guidelines for CPM

Many HCPs said their hospital did not have protocols, policies, or guidelines for CPM in place. Instead, they tended to subjectively assess and manage cancer pain without using standard pain rating scales or specific guidelines. Most doctors also relied on their personal experiences in oncology settings to assess and manage cancer pain:

*“As my duty, I always measure each patient's vital signs, including temperature, pulse rate, respiratory rate, and blood pressure. However, I never professionally measured the patient's pain level, as we do not have pain measurement tools in our clinic………….”* N5

*“We do not have guidelines for CPM. Accordingly, I usually assess and manage cancer pain based on my experience from the daily setting and the patient's facial expression…...”* D6

This could result in cancer patient’s pain may be under-measured and mismanaged due to a lack of pain rating scales and guidelines for CPM, which could result in inadequate CPM in Libya.

## Discussion

This study aimed to explore Libyan HCPs, patients, and caregivers' views and religious beliefs about cancer pain and its management. To the best of our knowledge, this is the first qualitative study to explore Libyan HCP, patient, and caregiver perceptions and beliefs about CPM. Factors that contribute to perceptions and beliefs of HCPs’, patients’, and caregivers’ attitudes to CPM include religion, culture, and economy, alongside the healthcare systems in Libya. The impact of these factors on HCP, patient, and caregiver perceptions and beliefs negatively impacts access to and use of appropriate CPM in Libya.

Similar to the findings of an earlier study (Hatamipour et al., 2015), many patients and their caregivers in the present study believed that Qur’an could cure diseases, such as cancer, and relieve physical suffering like pain. Although some studies with Muslim cancer patients show positive beliefs about their disease and pain (Hatamipour et al., 2015), these beliefs may influence CPM and can facilitate a reluctance to use medical treatment, such as opioids for CPM (Bosch & Baños, 2002). In this study, Libyan cancer patients and their caregivers relied on their religious beliefs, including belief and trust in Allah, reciting specific verses from the Qur’an, and prayer, to cope with cancer and pain. Although reciting verses from the Qur'an helped support Libyan patients, specifically with cancer pain, this reliance may negatively influence effective CPM in Libya, as some patients and their caregivers preferred to use the Qur'an instead of medications for CPM.

Specific verses are often used by patients seeking CPM. For example, this verse, “(*And We send down of the Qur’an that which is a cure and a mercy for the believers*)” (Verse 17:82 Surat AL-Isra) was commonly used by some cancer patients and their caregivers. However, it has been stated that the meaning of cure as expressed in this verse does not mean that the Qur’an cures the diseases like cancer and relieves physical pain, as some Muslims have thought. Rather than physical pain, the verse means that the Qur’an cures the spiritual diseases in people’s chests and hearts, such as jealousy, animosity, and hate (Attia, 2015). This could be why some patients and their caregivers refused to use opioids for CPM in the current study.

This study also illustrates that some Libyan HCPs described religious practices as applicable to support patients alongside medical intervention. This result confirms evidence, suggesting that HCPs should recognize and understand that patients often turn to their religious and spiritual beliefs when considering medical treatment (Dedeli & Kaptan, 2013; Swihart & Martin, 2020). Several studies showed that patients with pain, who were more likely to have better well-being psychologically and used positive coping strategies to cope with their suffering, were either religious or spiritual individuals (Baetz & Bowen, 2008; Dedeli & Kaptan, 2013). However, such coping strategies were related to better pain tolerance rather than pain relief (Baetz & Bowen, 2008). In the present study, some patients and their caregivers used religious beliefs as a coping strategy to tolerate pain; thus, they refused to use opioids for CPM.

This study confirms the findings with those of other studies (Kolmar & Kamal, 2018; Leong et al., 2016; Lunn, 2003), highlighting that cultural and religious beliefs could play an essential role in cancer patients' and caregivers’ attitudes towards CPM. A meta-analysis showed that cultural beliefs among Western and Asian patients were barriers to CPM (Chen et al., 2012). In the present study, some patients and their caregivers perceived using cautery as an alternative therapy to manage cancer pain. They believed it would work as it has a cultural perspective for treatment and is preferable to opioids for CPM. HCPs were aware of such cultural beliefs, but thought they had an adverse effect on CPM (Farid & El-Mansoury, 2015). Farid and El-Mansoury (2015) stated that due to the application of cautery by some Libyan patients to cure their cancer, cancer management is usually delayed, increasing the aggressiveness of the disease associated with chronic cancer pain (Farid & El-Mansoury, 2015). In the current study, patients’ and caregivers’ views about the use of cautery for CPM might be a barrier to effective CPM in Libya.

Although several guidelines have been established worldwide for effective CPM, such as World Health Organisation (WHO) (WHO, 2019) and National Institute for Health and Care Excellence (NICE) (NICE, 2012), in this study, Libyan HCPs frequently revealed that they did not follow any guidelines for CPM, including NICE and WHO for CPM because such guidelines do not exist in their clinics. To manage Libyan patients with cancer pain, most Libyan HCPs used textbooks and internet resources and their individual experiences of cancer care. HCPs from countries such as Canada, the USA (McCaffery & Ferrell, 1995), and the U.K. (Wells et al., 2002), who had the most extensive work experience in cancer and palliative care settings and followed specific guidelines for CPM, perhaps have more knowledge and positive attitudes towards CPM compared to HCPs from Libya. A possible explanation for this might be that Libyan HCPs do not have experience in palliative care settings, as palliative care does not exist in the Libyan healthcare system (El Ansary et al., 2014). A study found that HCPs with work experience in palliative care reported significant mean knowledge scores about CPM, *p* < 0.05 (Etafa et al., 2020).

Furthermore, Libyan HCPs do not refer to any guidelines for CPM, including NICE and WHO. Although Libyan HCPs had work experience in Libyan cancer care settings, experience in oncology units only may not be sufficient to improve CPM in Libya (Oldenmenger et al., 2009; WHO, 2019). Thus, in this study, cancer pain in Libya might be inadequately managed due to a lack of experience in palliative care, and specific guidelines for CPM, such as NICE and WHO, were not applied.

Although pain rating scales have been well-designed and used worldwide (BPS, 2019), such scales are not available for assessing cancer pain in Libya. Most Libyan HCPs said they relied on the patient's facial expression and self-report to assess cancer pain in the present study. It seems that an unprofessional assessment of cancer pain might impact CPM in Libya. Studies suggest that relying on HCPs’ assessments of facial expressions or patient self-reports is a significant barrier to effective CPM (Anderson et al., 2000). The lack of guidelines and pain rating scales for CPM means that cancer patients' pain may be under-measured and mismanaged, resulting in poor CPM in Libya.

A lack of knowledge and availability of CPM training for HCPs has been reported as significant barriers to effective CPM (Darawad et al., 2019). In this study, none of the HCPs had training in CPM, and they reported a lack of knowledge about CPM, resulting in some cases with cancer pain that might not have received adequate CPM. A cross-sectional survey conducted by Nasser et al. (2016), which included 69 Lebanese physicians, reported that about 57% of physicians were shown to have a very good to excellent level of knowledge and skills in CPM (Nasser et al., 2016). Although roughly 50% of Lebanese physicians recently had formal training in CPM, fear of side effects, tolerance, and addiction to opioids was still reported among Lebanese HCPs (45%) as the most common barrier to effective CPM (Nasser et al., 2016). However, this was more common among newly qualified physicians with fewer experiences in CPM (Nasser et al., 2016). In the current study, Libyan physicians reported that newly qualified doctors usually avoid prescribing opioids to patients for CPM as they were fearful of tolerance and addiction to opioids. Hence, it seems clear that positive attitudes and knowledge about cancer pain and opioids among HCPs are associated with higher professional education, training, and more extensive experience in cancer care and pain management settings. This view is consistent with Wells et al. (2002), who highlighted that improvement in HCPs' knowledge and attitudes about opioids for CPM following experience, professional teaching, and training in CPM (Wells et al., 2002). Another study reported that most physicians, who had more palliative care training, correctly answered 71% of questions related to knowledge about opioids and pain management (Rurup et al., 2010). In the present study, some Libyan doctors with long experience working in cancer care settings showed adequate knowledge and skills in CPM compared to newly qualified physicians. However, direct experience in oncology units without professional education and training in CPM is not enough to improve the knowledge of HCPs about CPM (Darawad et al., 2019). Hence, professional education and training (Hooten & Bruce, 2011) are needed to enhance CPM in Libya.

### Limitations

Although we sampled for maximum diversity, the range of perceptions that we found may not reflect the perceptions of HCPs, cancer patients, and caregivers in all parts of Libya because the inclusion of participants was from Eastern Libya only. Another limitation is that the focus of this study did not include the perceptions and views of hospital managers and policymakers about CPM. A survey conducted by Toba et al., (2019) reported that strict regulation on the use of opioids was perceived by approximately 70% of nurses as the most common barrier to CPM related to the healthcare system. Another study conducted in Thailand also found that among 47 policymakers, about 75% of them had inadequate knowledge, and 66% had negative attitudes towards opioids for CPM (Srisawang et al., 2013). Furthermore, the sample size might be considered small and may be seen as a study limitation. Nevertheless, in qualitative studies, the focus usually is on the quality of the data collected, including the credibility and the attainability within the available resources, but not on the study’s sample size (Patton, 2002).

## Implications for Practice and/or Research

The findings of this study have implications for HCPs' education and practice and healthcare policy. Our research on the perceptions and beliefs of Libyan HCPs, patients, and caregivers about CPM can contribute to the design of future interventions for CPM. Thus, improving and evaluating specific interventions that can be delivered briefly and easily in the Libyan context of CPM are needed (Bennett et al., 2009) to ensure such interventions meet Libyan patients’ and their caregivers’ needs and preferences for improving CPM in Libya (Latter et al., 2016). To improve CPM in Libya, specific guidelines, such as WHO (WHO, 2019) and NICE (NICE, 2012), and palliative care services should be adopted into the Libyan health system. Furthermore, a multidisciplinary team, including oncologists, clinical nurses, CPM specialists, psychiatrists, social workers, and religious scholars, should be located at each Libyan hospital and oncology centre.

Moreover, continuing professional education and training in CPM are needed to improve Libyan HCPs’ attitudes and knowledge about CPM. Additionally, involving patients, caregivers, and religious leaders in the education sessions on opioids and CPM is needed to enhance their attitudes and knowledge about cancer pain and opioids for CPM. Implications related to religion and culture, patients’ religious and cultural beliefs about cancer pain, and opioids should be understood by the HCPs (Orujlu et al., 2022). HCPs should educate patients and their caregivers to increase their knowledge and change their negative attitudes about cancer pain and opioids. For example, patients and their caregivers should know that the Qur'an and prayers cannot cure diseases like cancer and relieve physical suffering like pain. Patients and caregivers should believe that opioids are the most effective treatment recommended for CPM (WHO, 2019). For cultural implications, HCPs should educate patients and their caregivers about the misconceptions of cultural beliefs. For example, cancer management can be delayed due to the application of cautery by some patients to cure their cancer and pain, resulting in increasing the aggressiveness of the disease associated with chronic cancer pain (Farid & El-Mansoury, 2015).

For research implications, based on the findings of this study and due to Libyan HCPs, who participated in this study were only 12 participants (6 oncology nurses and 6 oncologists). Accordingly, further research with large sample size is required to fully understand contextual differences in Libya's current state of practice regarding CPM and HCPs’ attitudes and knowledge towards cancer pain and opioids for CPM in Libya.

## Conclusions and Recommendations

### Conclusions

This qualitative study shows that Libyan patients, caregivers, and HCPs hold negative CPM views due to religious and cultural beliefs. Furthermore, lack of knowledge, training in CPM, and experience in palliative care among Libyan HCPs might prevent effective CPM. Moreover, the economic and Libyan healthcare system-related factors are barriers to effective CPM. To address these concerns, developing and evaluating interventions, such as CPM education and training, would be necessary to improve patients’ outcomes with cancer pain in Libya.

### Recommendations for Future Research

One recommendation for future study can be that further research with other HCPs, including surgeons, anaesthesiologists, general practitioners (GP), hospital managers, and policymakers, is required. Another recommendation is that a study that includes participants from all parts of Libya is needed because the inclusion of participants in the current study was from Eastern Libya only.

## Supplementary Information

Below is the link to the electronic supplementary material.

Supplementary file1 (DOCX 27 KB)

## Data Availability

Data available on request from the authors: the data that support the findings of this study are available from the corresponding author, [SM], upon reasonable request.
