# Supplementary material for: Libyan Healthcare Professionals’, Patients’ and Caregivers’ Perceptions and Religious Beliefs about Cancer Pain and its Management: A Descriptive Qualitative Study
Source: J Relig Health. 2023 Feb 22;62(3):1897–919. doi: 10.1007/s10943-023-01763-1 (PMC10133376; doi:10.1007/s10943-023-01763-1)
Supplement: Supplementary file 1 — Supplementary file1 (DOCX 27 KB) [file 10943_2023_1763_MOESM1_ESM.docx]

Supplementary materials

# **Appendix 1 Questions for the interview with caregivers**

**Semi-structured interview guidelines and questions for family caregivers**

**N.B. This is a guide and the questions will vary depending on individual responses, which are family caregivers.**

This interview asks for your opinions and understanding of cancer pain and its management, and it will take about 30 to 45 minutes of duration.

**Interview questions for family caregivers**

Q 1. Can you tell me about yourself?

**Caregiver’s understanding of cancer pain and its management**.

Q 2. Can you tell me about your experience of cancer pain and how you have managed your family member's cancer pain?

**Caregiver’s perception of pain and opioids.**

Q 3. Can you tell me your thought on cancer pain?

PROBES:

a. Do you think cancer pain is part of disease progression?

b. Do you think cancer pain can be relieved?

Q 4. Can you tell me your opinion about opioids?

PROBES:

a. Do you think pain medicine can effectively control cancer pain?

b. Do you think there is a danger for the patient to become addicted to pain medicine?

c. Do you think opioid tolerance and/or addiction are the main reason for the patient to reject opioids for their pain?

d. Do you think opioid side effects are barriers to cancer pain management?

**Caregiver’s attitudes and knowledge towards cancer pain management.**

Q 5. Can you tell me what you usually do when your family member is having cancer pain?

PROBES:

a. Who do you talk to when the patient is having pain?

b. How do you manage his/her pain?

c. When does the patient last time asked for treatment for cancer pain? Why did he/she ask for it?

d. What kind of medications has the patient used before and now for the cancer

pain?

**Caregiver’s general views about cancer pain and its management.**

Q 6. How do you think your community perceives cancer pain management?

PROBES:

a. Do you think people more generally believe cancer pain should be managed?

b. Do you think religion and culture can influence in some way with cancer pain

management?

c. To what extent do these beliefs influence how you manage cancer pain?

Q 7. Do you think doctors usually focus on treating the disease, more than controlling pain?

Q 8. Do you think doctors prefer not to talk about cancer pain? If so, why?

Q 9. Do you think doctors might find it annoying to be told about pain?

Q 10. Do you think nurses prefer not to talk about cancer pain? If so, why?

Q 11. Do you think patients usually prefer not to talk about their pain? If so, why?

Q 12. Do you think doctors are influenced by their culture and beliefs when they are interpreting or managing your cancer pain?

Q 13. Do you think nurses are influenced by their culture and beliefs when they are interpreting or managing your cancer pain?

Q 14. Do you think patients are influenced by their culture and beliefs when they are interpreting or using medications for cancer pain?

Q 15. Can you tell me anything else about cancer pain and its management, which we did not cover during this interview?

Thank you for your participation in this interview

# **Appendix 2 Questions for the interview with patients**

**Semi-structured interview guidelines and questions for cancer patients**

**N.B. This is a guide, and the questions will vary depending on individual responses, which are cancer patients.**

This interview asks for your opinions and understanding of cancer pain and its management, and it will take about 30 to 45 minutes of duration.

**Interview questions for patients**

Q 1. Can you tell me about yourself?

**Patient’s understanding of cancer pain and its management.**

Q 2. Can you tell me about your experience of cancer pain and how you have managed your cancer pain?

**Patient’s perception of pain and opioids.**

Q 3. Can you tell me about your thought on cancer pain?

PROBES:

a. Do you think cancer pain is part of disease progression?

b. Do you think cancer pain can be relieved?

Q 4. Can you tell me your opinion about opioids?

PROBES:

a. Do you think pain medicine can effectively control your cancer pain?

b. Do you think there is a danger for you to becoming addicted to pain medicine?

c. Do you think opioid tolerance and/or addiction are the main reason for you to reject opioids for your pain?

d. Do you think opioid side effects are barriers to cancer pain management?

**Patient’s attitudes and knowledge towards cancer pain management.**

Q 5. Can you tell me what you usually do when you have cancer pain?

PROBES:

1. Who do you talk to when you have pain?
2. How do you manage your pain?
3. When do you last time asked for treatment for cancer pain? Why did you ask for it?
4. What kind of medications have you used before and now for cancer pain?

**Patient’s general views about cancer pain and its management.**

Q 6. How do you think your community perceives cancer pain management?

PROBES:

a. Do you think people more generally believe cancer pain should be managed?

b. Do you think religion and culture can influence in some way with cancer pain

management?

c. To what extent do these beliefs influence how you manage your cancer pain?

Q 7. Do you think doctors usually focus on treating the disease more than controlling pain?

Q 8. Do you think doctors usually prefer not to talk about cancer pain? If so, why?

Q 9. Do you think doctors might find it annoying to be told about pain?

Q 10. Do you think nurses usually prefer not to talk about cancer pain? If so, why?

Q 11. Do you think patients usually prefer not to talk about their pain? If so, why?

Q 12. Do you think doctors are influenced by their culture and beliefs when interpreting or managing your cancer pain?

Q 13. Do you think nurses are influenced by their culture and beliefs when interpreting or managing your cancer pain?

Q 14. Do you think family caregivers are influenced by their culture and beliefs when interpreting or using medications for your cancer pain?

Q 15. Can you tell me anything else about cancer pain and its management, which we did not cover during this interview?

Thank you for your participation in this interview

# **Appendix 3 Questions for the interview with HCPs**

**Semi-structured interview guidelines for oncology (physicians and nurses)**

**N.B. This is a guide, and the questions will vary depending on individual responses, which are oncologists and oncology nurses.**

This interview asks for your opinions and understanding of cancer pain and its management, and it will take about 30 to 45 minutes of duration.

**Interview questions for oncology physicians**

Q 1. Can you tell me about yourself?

**Oncologist’s understanding of cancer pain and its management.**

Q 2. Can you tell me about your experience with cancer pain and how you have managed your patients' cancer pain?

**Oncologist’s perception of pain and opioids.**

Q 3. Can you tell me about your thought on cancer pain?

PROBES:

a. Do you think cancer pain is part of disease progression?

b. Do you think cancer pain can be relieved?

Q 4. Can you tell me your opinion about opioids?

PROBES:

a. Do you think pain medicine can effectively control cancer pain?

b. Do you think there is a danger for the patient to become addicted to pain medicine?

c. Do you think opioid tolerance and/or addiction are the main reason the patient rejects opioids for her pain?

d. Do you think opioid side effects are barriers to cancer pain management?

**Oncologist’s attitudes and knowledge towards cancer pain management.**

Q 5. Can you tell me what you usually do when a patient has cancer pain?

PROBES:

a. How do you control cancer pain?

b. When does the patient last time asked for treatment for cancer pain? Why did he/she ask for it?

c. Do patients and their caregivers usually request a specific kind of medication for cancer pain management?

d. What kinds of medications have the patient used before and now for cancer pain?

e. Do you prescribe morphine for cancer pain management? If not, why not?

f. Do patients and their caregivers easily accept morphine for cancer pain management? If the answer is no, why not?

**Oncologists’ general views about cancer pain and its management.**

Q 6. How do you think your community perceives cancer pain management?

PROBES:

a. Do you think people more generally believe cancer pain should be managed?

b. Do you think religion and culture can influence in some way with cancer pain

management?

c. To what extent do these beliefs influence how you manage your patient’s cancer pain?

Q 7. Do you think doctors usually focus on treating the disease more than controlling pain?

Q 8. Do you think doctors usually prefer not to talk about cancer pain? If so, why?

Q 9. Do you think doctors might find it annoying to be told about pain?

Q 10. Do you think nurses usually prefer not to talk about cancer pain? If so, why?

Q 11. Do you think patients usually prefer not to talk about their pain? If so, why?

Q 12. Do you think doctors are influenced by their culture and beliefs when interpreting or managing your cancer pain?

Q 13. Do you think nurses are influenced by their culture and beliefs when interpreting or managing your cancer pain?

Q 14. Do you think caregivers are influenced by their culture and beliefs when interpreting or using medications for cancer pain?

Q 15. Do you think patients are influenced by their culture and beliefs when interpreting or using medications for cancer pain?

16. Do you think patients and their caregivers hold some concerns about cancer pain management?

Q 17. Do you think patients’ attitudes towards cancer pain management are influenced by their caregivers’ attitudes and the patient’s pain knowledge?

**Training and experience in cancer pain management.**

Q 18. What training or experience do you have in cancer pain management?

Q 19. From where do you learn about cancer pain and its management?

Q 20. Can you tell me anything else about cancer pain and its management, which we did not cover during this interview?

Thank you for your participation in this interview
